# Insights and Ideas Garnered from Marine Metabolites for Development of Dual-Function Acetylcholinesterase and Amyloid-β Aggregation Inhibitors

**DOI:** 10.3390/md12042114

**Published:** 2014-04-04

**Authors:** Shana V. Stoddard, Mark T. Hamann, Randy M. Wadkins

**Affiliations:** 1 Department of Chemistry and Biochemistry, University of Mississippi, 409 Coulter Hall, University, MS 38677, USA; E-Mail: shana.stoddard@stjude.org; 2 Department of Pharmacognosy, University of Mississippi, 407 Faser Hall, University, MS 38677, USA; E-Mail: mthamann@olemiss.edu

**Keywords:** molecular docking, enzyme inhibitor, sesquiterpene acetate, pyrrole, tetrazacyclopentazulene, bromotyrosine derivative, plastoquinone, farnesylacetone

## Abstract

Due to the diversity of biological activities that can be found in aquatic ecosystems, marine metabolites have been an active area of drug discovery for the last 30 years. Marine metabolites have been found to inhibit a number of enzymes important in the treatment of human disease. Here, we focus on marine metabolites that inhibit the enzyme acetylcholinesterase, which is the cellular target for treatment of early-stage Alzheimer’s disease. Currently, development of anticholinesterase drugs with improved potency, and drugs that act as dual acetylcholinesterase and amyloid-β aggregation inhibitors, are being sought to treat Alzheimer’s disease. Seven classes of marine metabolites are reported to possess anti-cholinesterase activity. We compared these metabolites to clinically-used acetylcholinesterase inhibitors having known mechanisms of inhibition. We performed a docking simulation and compared them to published experimental data for each metabolite to determine the most likely mechanism of inhibition for each class of marine inhibitor. Our results indicate that several marine metabolites bind to regions of the acetylcholinesterase active site that are not bound by the clinically-used drugs rivastigmine, galanthamine, donepezil, or tacrine. We use the novel poses adopted for computational drug design of tighter binding anticholinesterase drugs likely to act as inhibitors of both acetylcholinesterase activity and amyloid-β aggregation inhibition.

## 1. Introduction to Acetylcholinesterase Structure and Function

Acetylcholinesterase (AChE) is a member of the α/β hydrolase fold family of enzymes [1]. This enzyme degrades the excitatory neurotransmitter acetylcholine (ACh) in the synaptic junction at an extraordinarily fast catalytic rate, with a 2nd order rate constant almost as fast as a diffusion-controlled reaction [2,3]. ACh is degraded to choline and acetate through a hydrolysis mechanism, resulting in decreased signal transmission in nerve synapses.

In 1991, using X-ray crystallography, Sussman’s lab elucidated a 2.8 Å resolution structure of AChE from the *Torpedo californica* electric ray [4]. Two sites participate in the hydrolysis reaction of ACh: an anionic site and an esteratic site. The anionic site draws ACh into the active site, followed by hydrolysis in the esteratic site. The catalytic triad (Ser-200, Glu-327 and, His-440) lie at the bottom of a 20 Å gorge. This long, narrow gorge contains 14 conserved aromatic residues (e.g., Tyr-70, Trp-84, Tyr-121, Trp-279, Phe-288, Phe-290, Phe-330, and Tyr-334) leading to the active site [5]. Residues Phe-288 and Phe-290 and the catalytic triad create the esteratic site. Residues Trp-84 and Phe-330 create the anionic site [5]. Approximately 14 Å away from the anionic site is another negatively charged site called the peripheral anionic binding site (PAS), composed of residues Tyr-70, Asp-72, Tyr-121, Trp-279, and Tyr-334. Binding of substrates and inhibitors to the PAS causes a conformational change to AChE, reducing ACh’s ability to enter the active site [5,6].

Acetylcholinesterase is the drug target for treating the neural degenerative disorder Alzheimer’s disease (AD). AD in elderly individuals is characterized by memory loss, difficulty in storing new information, and behavioral and cognitive difficulties [7,8]. The progressive nature of AD can require a high level of care since patients lose the ability to perform simple daily functions.

There are two hypotheses to explain the pathology of AD. One suggests that the decrease in ACh production within the synaptic junction contributes to the onset of AD (cholinergic hypothesis) [9,10,11]. The other suggests that the development of toxic amyloid-β peptide aggregates in the brain contributes to the progression of AD (Amyloid hypothesis) [9,12]. The cholinergic hypothesis suggests that inhibition of AChE can result in improved cognition by increasing ACh activity. The amyloid hypothesis suggests that drugs that inhibit amyloid plaque formation will slow the progression of AD. Inestrosa demonstrated [13] that the PAS of AChE forms stable complexes with senile plaques promoting the formation of amyloid-β peptide aggregates, and that compounds that bind to the PAS of AChE can act as amyloid-β aggregation inhibitors. Therefore, some AChE inhibitors (AChE-I) have been shown to effectively prevent both ACh hydrolysis and plaque aggregation in AD. These dual-function inhibitors (DFI) have the potential to be more effective than single-function inhibitors.

**Figure 1:**
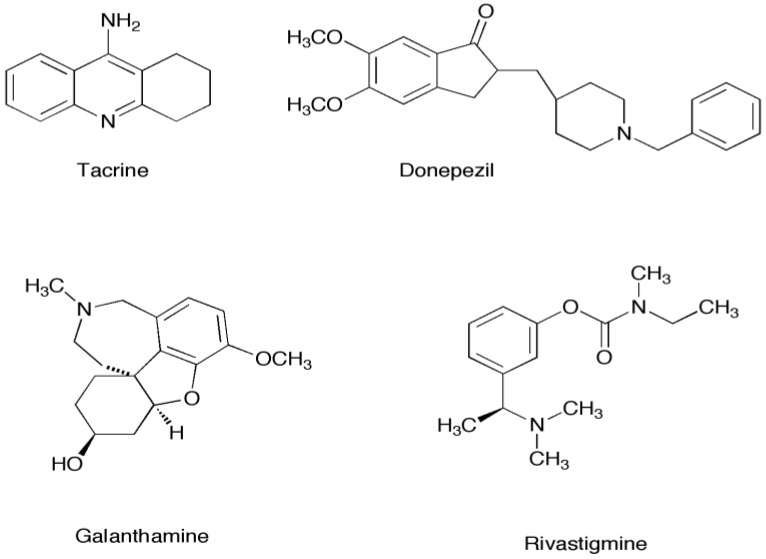
Current clinically-used acetylcholinesterase inhibitors.

Current clinical AD therapies use the anticholinesterase drugs rivastigmine, tacrine, galanthamine, and donepezil [11,14]. (Figure 1) Binding modes of these drugs are depicted in Supplementary Figure S1. The inhibition of AChE increases the amount, and prolongs the duration, of ACh present in the synaptic junction. More ACh is then allowed to enter the nicotinic receptors due to increased ACh levels. The current chemotherapeutic options have low specificity toward AChE and can be poorly tolerated by patients [10]. Patients receiving donepezil show only moderate improvement of symptoms of AD [9,14]. Thus development of higher affinity inhibitors may also help to alleviate the mental impairment associated with AD. Recently, inhibitors that inhibit both AChE and prevent amyloid-β aggregation have been suggested as a new therapeutic route [15,16,17,18], although there are none currently in use.

### 1.1. Marine Metabolites as Acetylcholinesterase Inhibitors

Presently, there are no marine natural products in clinical use as AChE-I. Given the past success of drugs derived from marine organisms [19], exploring marine metabolites (MM) for novel lead anticholinesterase compounds may identify new compounds with novel interactions with AChE that garner selectivity and gain potency in treating AD. The purpose of this article, then, is the comparison of known marine-derived compounds having anticholinesterase activity to compounds whose mechanism of action are well understood to identify both similarities as well as novel properties of the marine compounds.

Marine metabolites vary greatly in structure, mass, and chemical composition [20]. Only 7 different classes of MM are reported to have anticholinesterase activity: a sesquiterpene acetate [21,22], a pyrrole derivative [23,24], a tetrazacyclopentazulene [25], a bromotyrosine derivative [26,27], plastoquinones [28], farnesylacetones [28,29], and poly-alkylpyridinium polymers (Poly-APS) [30,31,32]. Overall, these metabolites have been found to have moderate levels of anticholinesterase activity and generate inhibition through several different mechanisms. A review by Hostettmann was published in 2006 on all acetylcholinesterase inhibitors known up to that point, including those of marine origin [33]. Our paper includes new inhibitors discovered from marine species since 2006. Hostettmann’s review detailed the structural information but not the enzyme-inhibitor interactions. Our focus is illustrating the inhibitor interactions within the AChE receptor and comparing them to compounds with known inhibition mechanisms. We have incorporated both experimental studies from the literature and our molecular ensemble docking studies of MM into the AChE receptor to better understand the molecular interactions of each inhibitor class, and propose novel hybrid compounds that would combine the best of both synthetic and MM compounds.

## 2. Results and Discussion

### 2.1. The Opistobranch Mollusk and its Metabolite, Onchidal: A Sesquiterpene Acetate

**Figure 2:**
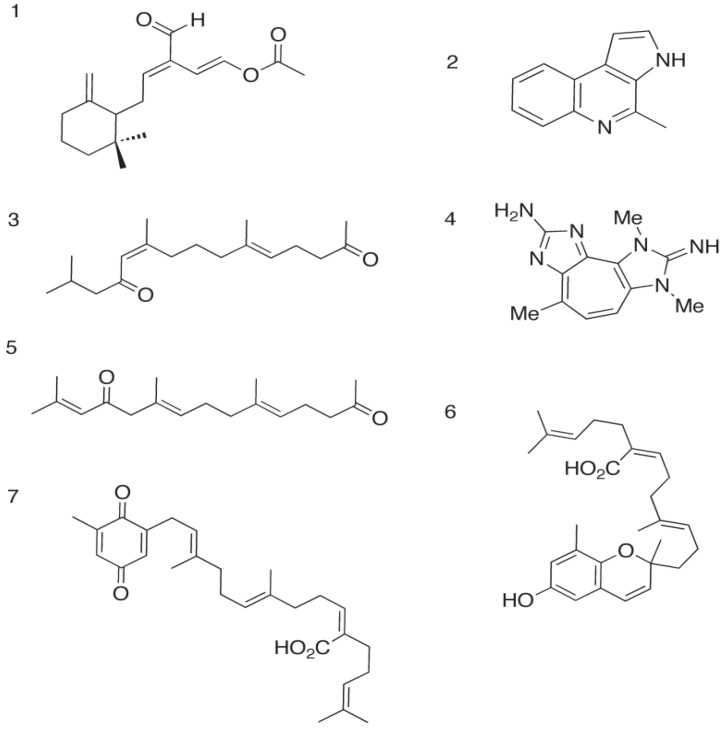
Marine metabolites that inhibit acetylcholinesterase Marine metabolites with anticholinesterase activity: **1**. Onchidal [20,21]; **2**. Marinoquinoline [22,23]; **3**. Dihydromonofarnesylacetone [27,28]; **4**. Pseudozoanthoxanthin-like compound (PZT) [24]; **5**. Monooxofarnesylacetone [27,28]; **6**. Sargachromenol [27]; **7**. Sargaquinoic acid [27].

In 1978, the compound onchidal (Figure 2), a sesquiterpene acetate containing an α/β-unsaturated aldehyde and an acetate ester, was isolated from the mucous secretion of the *O. binneyi* mollusk species by Ireland and Faulkner [21]. Onchidal was shown to interact with the acetylcholine recognition site, specifically the esteratic site, and found to have a *K_d_* of 300 μM.

#### 2.1.1. Docking of Onchidal into AChE

**Figure 3:**
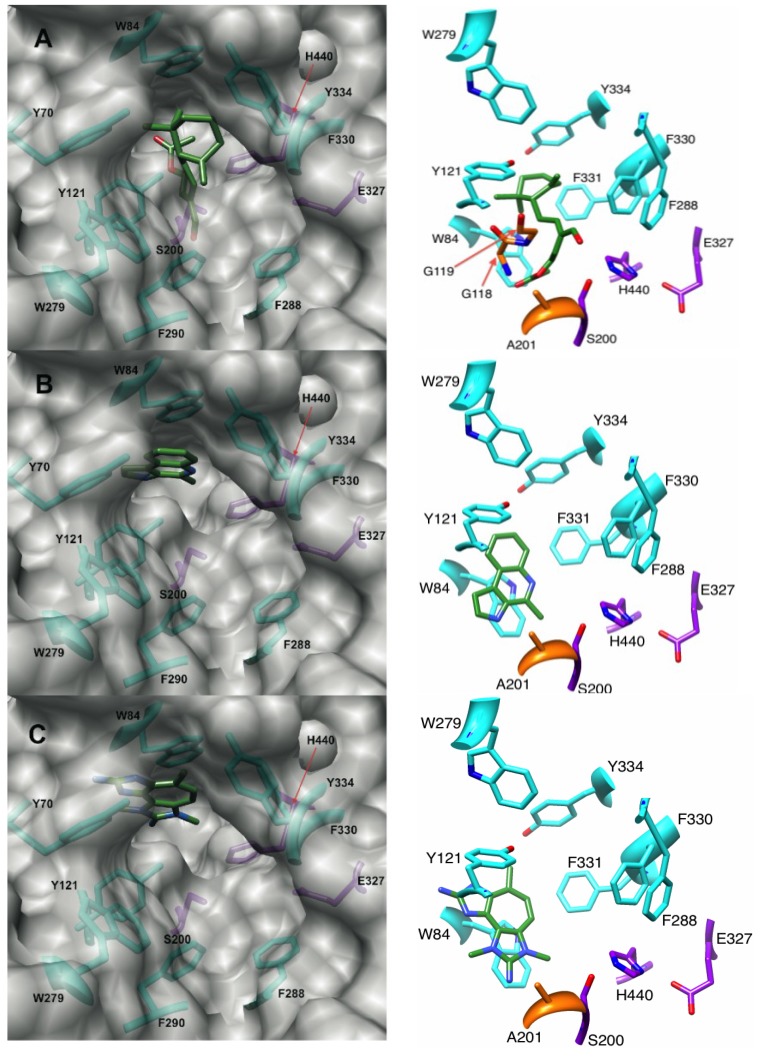
Binding modes and molecular interactions of onchidal, marinoquinoline and PZT compound metabolites to AChE. Docking poses of (**A**) Onchidal; (**B**) Marinoquinoline; and (**C**) PZT compound in the acetylcholinesterase receptor. Left panel orientation: looking into the active site. Right panel orientation: 90° upward rotation from left panel. Purple, orange, and cyan residues are catalytic triad, oxyanion hole, and aromatic gorge residues respectively.

In our docking experiments, onchidal was shown to interact with the esteratic site of AChE, which is consistent with Abramson’s work [22]. Three poses were generated for onchidal (Supplementary Figure S2). In all three poses the cyclohexane ring is positioned in the bottleneck between the surface of AChE and the active site residues. In two of the three poses, the acetate ester is in the oxyanion hole. The lowest energy pose (Figure 3A) for onchidal was generated from the 1DX6 receptor and places the acetate ester in the oxyanion hole, the aldehyde in the acyl pocket and onchidal’s cyclohexane ring in the bottleneck created by Phe-330 and Tyr-121. The major interactions were potential hydrogen bonding (H-bonding) interactions with Gly-118 and Gly-119 of the oxyanion hole, hydrophobic contacts with the bottleneck, and a possible H-bond with His-440 of the catalytic triad (Figure 3A).

During catalysis by AChE, H-bonding to the backbone residues Gly-118, Gly-119, and Ala-201 stabilizes the tetrahedral intermediate formed in the substrate. This suggests compounds capable of H-bonding with the oxyanion hole could inhibit AChE in a novel manner.

Some aldehyde protease inhibitors have been shown to interact with the oxyanion hole and provide strong inhibition [34,35] of these enzymes. The potential H-bonding interaction with oxyanion hole residues in AChE is unique to marine metabolites *vs.* synthetics; interactions with the oxyanion hole and His-440 are not possible when donepezil, galanthamine, tacrine and rivastigmine are docked into AChE. The hydroxyl group of galanthamine does interact with a water molecule that is located in the oxyanion hole, but not the residues of the oxyanion hole itself [36].

When onchidal is docked in the 1DX6 receptor with this conserved water molecule present, it resulted in onchidal having a clockwise rotation to prevent steric clashes with the water molecule (Supplementary Figure S3). The ester group shifts backwards 2.96 Å away from the catalytic serine, and the aldehyde group moves 6.34 Å downward. This may be the initial binding mode onchidal undergoes, after which onchidal may rotate displacing the water in oxyanion hole. Because onchidal is an irreversible inhibitor, a covalent bond is formed between it and the AChE receptor. Several reaction mechanisms for onchidal inhibition were suggested by Abramson [22]: (i) Schiff base formation with a lysine; (ii) attack on the beta-unsaturated carbon in conjugation with the aldehydes by a Cys, Lys, His, or Tyr; and (iii) the formation of 1,4-dialdehyde, which reacts with a Lys to form a pyrrole covalent adduct. Abramson did not have the benefit of the crystal structure of AChE solved by Sussman in 1991, limiting their mechanistic analysis. All Lys in AChE are located on the outside and not buried inside. There are 7 Cys in AChE, six of which are paired and involved in intrachain di-sulfide bonds, and the last is not near the active site or in the gorge. Only one His, the active site His-440, is located in the gorge. Several Tyr are located in the gorge; Tyr 442, 334, 130, 121, and 70. Based on the crystal structure and Abramson’s findings, the most probable mechanism is attack of the beta-unsaturated carbon in conjugation with the aldehyde by a Tyr; however a candidate Tyr for this mechanism was not identified from our docking analysis. In any event, onchidal’s irreversible inhibition of AChE makes it unsuitable for direct use as an anticholinesterase inhibitor for human diseases, since permanent inhibition of AChE leads to potentially deadly cholinergic toxicity, but it could have potential for use in insecticides or pesticides. Onchidal’s interaction with the oxyanion hole is a new mechanistic area for incorporation into rational drug design of novel inhibitors (see below).

### 2.2. The Gliding Bacteria Rapidithrix thailandica and Its Pyrrole Metabolites

*Rapidithrix thailandica* is a gliding bacterium that was discovered in seaweed extracted from the Andaman Sea off the coast of Thailand by Srisukchayakul [37,38]. Kanjana-opas and co-workers [23] isolated pyrrole derivatives marinoquinoline (Figure 2), 3-(2′aminophenyl)-pyrrole, and 2,2-dimethyl-pyrrolo-1,2-dihydroquinoline, from *R. thailandica* in 2006. Marinoquinoline, which has an extended aromatic system, inhibited AChE with an IC_50_ value of 4.9 μM. The other isolated metabolites did not possess anticholinesterase activity. The inactive pyrrole derivatives isolated lacked an extensive aromatic ring system. This structural difference suggested that inhibition produced by marinoquinoline was due to π–π stacking interactions.

#### 2.2.1. Docking of Marinoquinoline to AChE

**Figure 4:**
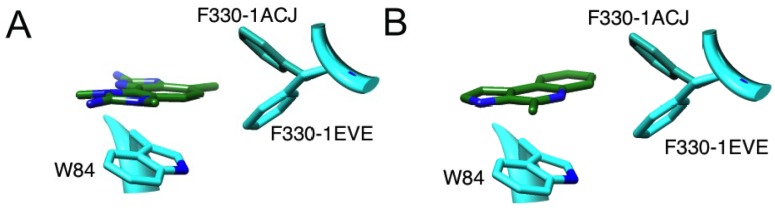
Molecular interactions of (**A**) marinoquinoline and (**B**) PZT-compound with 1ACJ and 1EVE receptors. Best docking score was shown with 1ACJ receptor, suggesting F330 would rotate to create double stacking interaction.

No prior work has elucidated which specific residue or residues in the aromatic gorge that marinoquinoline is potentially stacking against. We hypothesized that marinoquinoline interacts with AChE by participating in π–π stacking with Trp-84, similar to tacrine’s mode of binding [5]. We observed marinoquinoline docked against the Trp-84 (Figure 3B) in all low-energy structures with AChE, supporting the hypothesis of π–π stacking to an aromatic residue. In the 1ACJ structure that was crystallized with tacrine as a ligand, Phe-330 is oriented approximately 90° upward compared to its position in the 1ACL, 1DX6 and 1EVE structures (Supplementary Figure S4). Upon binding by tacrine, this Phe-330 forms a π-stacking interaction with the inhibitor. Docking marinoquinoline into the 1ACJ structure generated an improved score of −34.27 kJ/mol *vs.* the other three AChE receptor structures (Table 1), further suggesting that binding of marinoquinoline is analogous to that for tacrine (Figure 4A).

**Table 1:** Docking scores of current anticholinesterase drugs and marine metabolites into acetylcholinesterase receptors.

Receptor					
Ligand	1ACL	1ACJ	1DX6	1DX6 w/H_2_O	1EVE
	All Scores in kJ/mol				
Donepezil	−59.79	−10.86	−53.65	−61.18	−56.74
Tacrine	−35.28	−33.83	−33.91	−34.09	−34.36
Galanthamine	−35.28	−35.77	−36.47	−38.76	−36.38
Acetylcholine	−31.78	−34.06	−31.99	−31.25	−31.61
Onchidal	−47.51	−44.28	−48.55	−47.37	−46.14
Marinoquinoline	−30.77	−34.27	−31.09	−30.50	−31.05
Tetracyclopentazulene	−42.34 ^1^	−44.03 ^1^	−41.77 ^2^	−42.03 ^1^	−42.51 ^2^
Sargaquinoic acid	−67.92	ND	−63.14	−69.81	−67.15
Sargachromenol	−62.98	−61.45	−62.82	−67.67	−65.16
Monooxofarnesylacetone	−59.00	−55.34	−57.81	−50.22	−57.36
Dihydromonooxofarnesylacetone	−56.00	−55.38	−55.94	−52.83	−55.51

Docking galanthamine into its own receptor with and without this conserved water molecule both resulted in the crystallographic pose being selected. All inhibitors generated similar scores and poses in each receptor with the exception of donepezil in the 1ACJ receptor and onchidal. The best pose for onchidal was chosen. ^1,2^ Tetrazacyclopentazulene has two main poses (pose 1 scores are denoted by (^1^), pose 2 scores are denoted by (^2^)). ND Sargaquinoic acid would not dock into the 1ACJ receptor due to the Phe-330 ring position occluding the active site gorge (see Supplementary Figure S4).

### 2.3. The Parazoanthus axinellae (O. Schmidt), Zoanthid Corals, and the Tetrazacyclopentazulene Natural Products

The pigmentation of Zoanthid corals is vast, and produced by tetrazacyclopentazulene compounds (TCP; Figure 2). One yellow TCP, called a pseudozoanthoxanthin-like compound (PZT), was isolated by Turk and co-workers in 1994 [25]. Like onchidal, this compound was shown to be a competitive acetylcholinesterase inhibitor (*K_i_* of 4 μM). Unlike onchidal, which interacts with the esteratic site of acetylcholinesterase, the PZT compound was found to interact with the aromatic residues lining the active site gorge as determined by fluorescence emission spectroscopy. Turk and co-workers found that the signal from the intrinsic tryptophan located in the gorge of acetylcholinesterase was quenched in the presence of the inhibitor. These results suggested that an interaction between a Trp residue and the inhibitor was the mechanism of inhibition.

#### 2.3.1. Binding of a Tetrazacyclopentazulene (PZT) Compound to AChE

**Figure 5:**
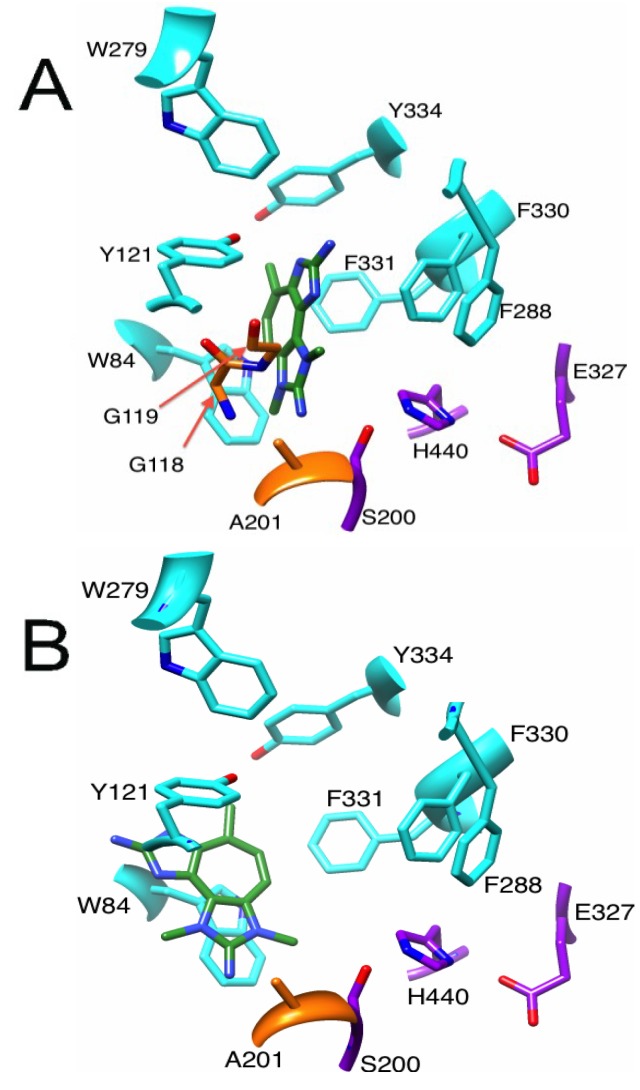
Poses generated from docking PZT-compound in the (**A**) 1EVE receptor and (**B**) 1ACL receptor.

Using the 1EVE receptor, the pose for the PZT-compound scored at −42.51 kJ/mol and showed orientation consistent with T-stacking with Trp-84. There is also a potential H-bonding interactions with the oxyanion hole residue Gly-118 (Figure 5A). The docking of the PZT compound into the 1ACL receptor resulted in a score of −42.34 kJ/mol, which positioned the PZT compound stacked against Trp-84 (Figure 5B). Both poses are in agreement with published experimental data that suggest interaction with a Trp residue. While Turk and coworkers could not determine which Trp residue PZT interacted with, our docking analysis suggest that Trp-84 is the most likely. There are also two other conserved Trp residues in the gorge: Trp-233, and Trp-279. Trp-279 is located at the entrance, while Trp-233 is in the acyl pocket. However, our docking analyses indicate that close association with Trp-279 and/or Trp-233 and the PZT compound is a higher energy conformer than that formed with Trp-84.

A PZT-compound-Trp-84 interaction likely shares the same binding mode as tacrine [5] and the one we propose for marinoquinoline. Again, a rotated Phe-330 would allow for double π–π stacking interactions (Figure 4B). Like marinoquinoline, the binding score improved when docked into the 1ACJ receptor (−44.04 kJ/mol). Thus, PZT most likely forms π–π interactions with both Trp-84 and Phe-330, which in turn closes off the bottleneck, preventing ACh from entering the acyl pocket. Tacrine’s best docking score was −35.28 kJ/mol, which is 9 kJ/mol weaker then PZT-compound binding score. A paper by Muñoz-Ruiz [18] used the indole ring of donepezil and the fused ring of tacrine to develop potent dual site AChE inhibitors, with the tacrine moiety bound to the anionic site. Replacement of the tacrine moiety by the PZT-compound during rational drug design has the potential to increase the potency of dual inhibitors even more (see below).

### 2.4. The Brown Alga Sargassum Sagamianum and the Plastoquinones and Farnesylacetones Metabolites

Ryu [29] in 2003, and Choi [28] and co-workers in 2007 reported the isolation of a series of terpenoid compounds (Figure 2) from the brown alga *Sargussum sagamianum.* Four compounds were isolated from the alga: sargaquinoic acid (IC_50_ of 23.2 μM), sargachromenol (IC_50_ of 32.7 μM), monooxofarnesylacetone (IC_50_ of 65.0 μM) and dihydromonooxofarnesylacetone (IC_50_ of 48.0 μM). All four compounds have moderate levels of bovine erythrocyte AChE inhibition, with a specificity for butyrylcholinesterase.

Based solely on structural comparisons, we hypothesized these compounds likely inhibit AChE in two ways: stacking interactions with aromatic residues, and interactions with the esteratic site. To date, no experimental work has been done to probe the mechanism of inhibition of the plastoquinones or farnesylacetones.

#### 2.4.1. Docking of the Plastoquinones and Farnesylacetones into AChE

**Figure 6:**
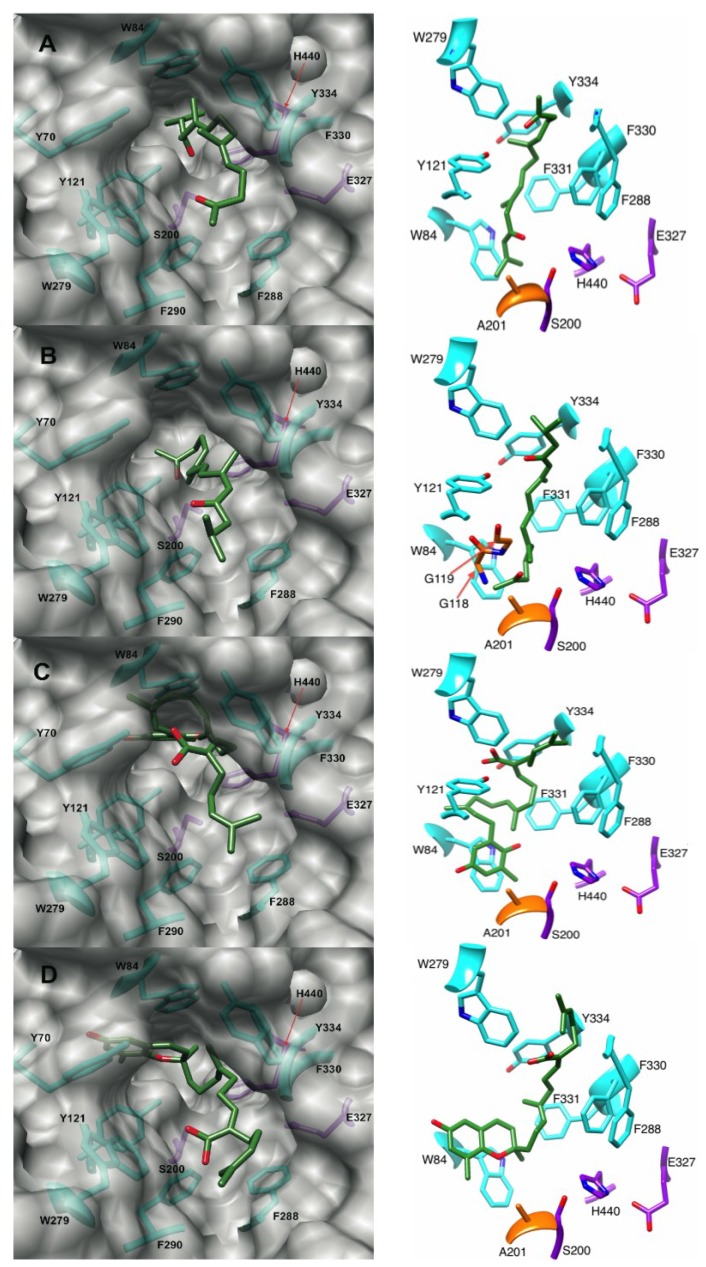
Poses of (**A**) monooxofarnesylacetone; (**B**) dihydromonooxofarnesylacetone; (**C**) sargaquinoic acid; and (**D**) sargachromenol docked into 1ACL, 1DX6, 1ACL, 1EVE AChE receptors respectively. Left panel: active site view. Right panel: 90° upward rotation from left panel. Purple, orange, and cyan residues are catalytic triad, oxyanion hole, and aromatic gorge residues respectively.

Docking of the two plastoquinones resulted in similar binding modes (Figure 6). Three stacking interactions were observed with the docked sargaquinoic acid: (i) the quinone moiety of sargaquinoic acid is positioned next to Trp-84; (ii) the 6–7 olefin is stacked with Phe-330; and (iii) the 10–11 olefin stacked against Tyr-334. Sargaquinoic acid’s binding score was the best of all metabolites considered, being −67.92 kJ/mol in the 1ACL receptor. Sargachromenol has one less stacking interaction in the docked structure than does sargaquinoic acid. We observed the reduced quinone moiety of sargachromenol stacking against Trp-84 and the 3–4 double bond of sargachromenol stacked against Phe-330 (Figure 6). Sargachromenol binds slightly weaker to the receptor than sargaquinoic acid, with its best binding score being −65.16 kJ/mol in the 1EVE receptor.

The farnesylacetones, unlike the plastoquinones, do not share a similar binding mode. Dihydromonooxofarnesylacetone has a binding score of −56.00 kJ/mol in the 1ACL receptor. In the oxyanion hole, its ketone moiety has potential to H-bond with Gly-119, and in the PAS, its α/β unsaturated ketone is positioned for a H-bonds to Tyr-121 (Figure 6). This compound is unique for this family in that it has greatly reduced stacking interactions. However, like sargaquinoic acid, monooxofarnesylacetone π-stacks against Trp-84, Phe-330 and Tyr-334 in the docked structure (Figure 6A). The binding score for monooxofarnesylacetone is almost 9 kJs weaker than sargaquinoic acid at −59.00 kJ/mol in the 1ACL receptor.

Both the plastoquinones and farnesylacetones are most likely competitive inhibitors of AChE, which occlude the active site gorge in a manner similar to donepezil. They all protrude somewhat out of the gorge, suggesting that they prevent acetylcholine from entering the AChE binding site. Donepezil stacks against Phe-330 and Trp-84 and interacts with the Trp-279 located in the PAS. The plastoquinones and monooxofarnesylacetone, like donepezil, primarily use stacking interactions to stabilize the receptor ligand complex, stacking against Trp-84, Tyr-334, and Phe-330 within the gorge. The isoprene units of the farnesylacetones and the plastoquinones have the right number of linker carbons and degree of flexibility to allow participation in multiple aromatic interactions simultaneously. In general, the plastoquinones and farnesylacetones bind to the side opposite the PAS at the entrance of the gorge, with the exception of dihydromonooxofarnesylacetone that H-bonds to Tyr-121 of the PAS.

In the development of dual binding site inhibitors that bind to the anionic site and to the peripheral anionic site, a linker is needed that can span the length of the gorge. Linkers that bind strongly to the gorge would greatly increase the potency of these dual site inhibitors. Many linkers used thus far have contained amides [15,18], aliphatic chains [16,18], pyrimidine rings [17], piperidine rings [15,16], and other cyclic aromatic structures [15,16]. In our docking studies, the plastoquinones bind tighter to AChE than donepezil (best score −59.79 kJ/mol) with farnesylacetones coming in close behind donepezil. The terpene type linker seems to bind well in the gorge, capitalizing on multiple stacking interactions. Thus, it may be a useful new linker to consider in creation of dual site inhibitors. Dihydromonooxofarnesylacetone has two ketone residues, one that binds the oxyanion hole and one that binds to the PAS residue Tyr-121, and therefore suggests a new di-ketone scaffold for amyloid-β.

### 2.5. Design of New Dual AChE and Amyloid-β Aggregation Inhibitors

**Figure 7:**
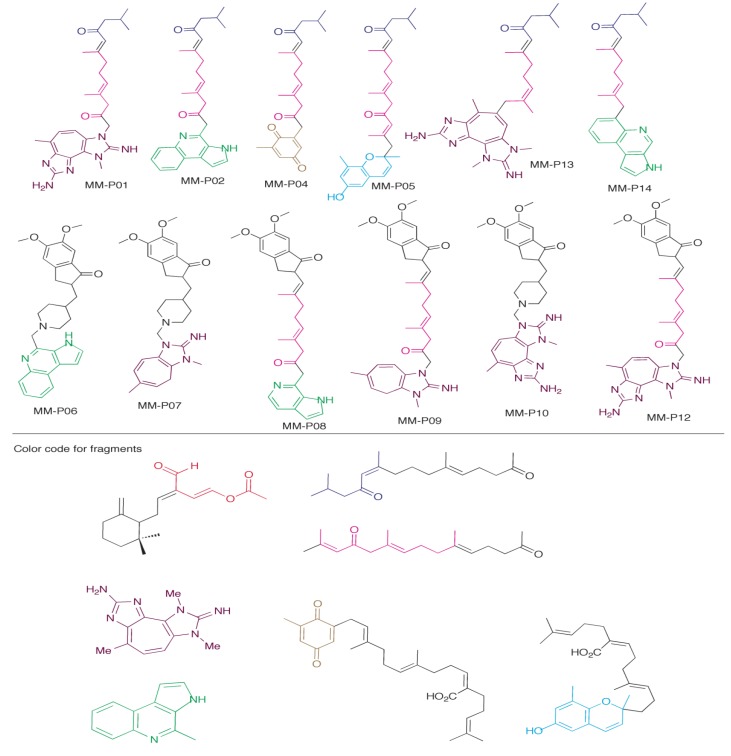
Proposed dual function inhibitors developed using marine metabolites (MM) scaffolds. Each compound was made using portions of MMs that had specific interactions within an AChE receptor. The indoline and piperidino moieties used in some of the compounds are from donepezil.

Using insights from the docking scores and poses of marine metabolites above, we propose 12 new AChE inhibitor candidates (Figure 7). We hypothesized that we could both generate new compounds using the MM as scaffolds that would have better binding scores and that we would be able to predict the pose of these compounds based on the docking data of the MM precursors used. These compounds were then docked into the 1EVE and 1DX6 receptors. Table 2 shows the docking scores of the designed inhibitor candidates. Eleven of the twelve compounds show improved docking scores compared to donepezil. Only one compound MM-P07 (best dock score −58.26 kJ) showed less favorable binding to the AChE receptor than donepezil. The best compound MM-P12 had a PZT base, a terpene linker, and the indoline ring of donepezil at the top. All of these inhibitor candidates have CLogP scores (calculated with ChemBioDraw 13) between 3.80 and 6.62. For comparison, the CLogP value for the clinically-used drug donepezil is 4.60, indicating that penetration of the blood-brain barrier by the candidates would similar.

While using the marine metabolites as starting point to develop new AChE inhibitor scaffolds with improved docking scores was successful, it was difficult to accurately predict the interactions they would have with the receptor. We found that several new interactions were employed in our candidate compounds. For example, Tyr-130 was not found to have any major interactions with the marine metabolites themselves. The best inhibitor candidate, MM-P12 (shown in Figure 7; docking score of −73.43 kJs) has an amino group, from the PZT compound scaffold that may potentially hydrogen bond with Tyr-130. Compounds MM-P04 (quinone moiety from sargaquinoic acid), MM-P05 (quinone moiety from sargachromenol), MM-P08 (indoline ring of donepezil) also were found to have potential H-bonds to with Tyr-130. We will not discuss the poses of each new inhibitor in detail but will highlight some important features contributing the increased potency of these compounds. We also observed that the orientation of compounds could be exactly opposite what we hypothesized for example. The PZT-compound and marinoquinoline were used in our scaffolds in an effort to drive what we considered the base of the compound to the anionic site through a stacking interaction with Trp-84 that was observed with each of the precursors.

The addition of the PZT compound to the terpenoid scaffold produced poses where this moiety was stacking against Trp-84 as expected, but also produced poses where it was located at the periphery of the gorge, participating in stacking reactions with Trp-279 in the PAS, and with Tyr-334. This produces a slightly more favorable dock score for the latter pose compared to the former (−72.93 kJ, *versus* −71.97 kJ). Both poses show improved binding to the AChE receptor compared to the MM precursors. This inversion of orientation was also observed for compounds MM-P08, MM-P07, and MM-P10 (1DX6 receptor only). While some new interactions were observed, most of the candidates adopted the expected poses.

**Table 2:** Docking scores of proposed dual function inhibitors into acetylcholinesterase receptors.

Compound	Best Score (kJ/mol)	Receptor	Interacts with PAS	MM Scaffolds Used to Create Compound (Figure 2)	CLogP
MM-P01	−72.93	1EVE	Yes	3, 4, 5	4.60
MM-P02	−63.02	1EVE	No	2, 3, 5	5.23
MM-P04	−62.62	1EVE	Yes	3, 5, 7	3.81
MM-P05	−71.95	1EVE	Yes	3, 5, 6	6.62
MM-P06	−62.00	1DX6	Yes	2, and donepezil	5.01
MM-P07	−58.26	1DX6	Yes	4 and donepezil	4.68
MM-P08	−65.76	1EVE	Yes	2, 5, and donepezil	4.78
MM-P09	−72.19	1EVE	Yes	4, 5, and donepezil	5.98
MM-P10	−64.77	1DX6	Yes	4 and donepezil	4.24
MM-P12	−73.43	1EVE	No	4, 5, and donepezil	5.54
MM-P13	−68.90	1DX6	No	3, 4, 5	4.93
MM-P14	−60.78	1EVE	No	2, 3, 5	6.24

Dual function inhibitors must interact with the PAS of AChE to be effective. Of the 12 compounds we designed, eight have the capability of interaction with the PAS in some fashion (MM-P01, MM-P04, MM-P05, MM-P06, MM-P07, MM-P08, MM-P09, and MM-P10). Four of these compounds stack with Trp-279 (MM-P06, MM-P07, MM-P09, and MM-P10). MM-P04, MM-P05 also have potential H-bonds to Tyr-121. Two compounds interact with both residues (MM-P01, and MM-P08). Overall, the insights obtained from out docking analysis of marine metabolites into AChE has provided ideas for several candidate structures of potential dual function inhibitors that exhibit properties likely to make them better anti-Alzheimer’s drugs than those currently in clinical use.

## 3. Experimental Section

Computational evaluation can delineate important interactions between the inhibitors and receptors, where mechanisms of inhibition and sites of interactions are unknown. Docking is a technique that has been performed for many biologically-important receptors [39,40]. It is used here to predict the interactions between AChE and an inhibitor, suggesting the inhibitor’s binding mode(s).

Using the Marvin Sketch program (Chem Axon), compounds were converted from 2D to 3D structures, after which an initial structural energy minimization was performed. Chimera (UCSF [41]) was then used to perform a full structural minimization (using steepest decent followed by conjugate gradient), to calculate total charges on the molecules, and to add all remaining hydrogens not explicitly defined in the 2D structure. DOCK 6.3 was then used for all docking analyses. The success rate for DOCK (70%) is comparable to FlexX (61%), Glide (82%), and GOLD (77%) [42,43]. Default parameters were used throughout the flexible docking analysis, with the exception of number of orientations, which was varied through the course of several docking simulations (1000 to 30,000 orientations evaluated). When the docking simulation did not locate a minimum for a particular compound (incomplete docking), the orientation number was increased for the dataset. Four acetylcholinesterase structures (pdb codes 1ACL, 1DX6, 1EVE and 1ACJ) were used for all docking analysis. The active site was identified using the ligands galanthamine, donepezil and tacrine located in the crystal structures of 1DX6, 1EVE and 1ACJ, respectively. For the 1ACL, to define the active site with a clinically-used inhibitor, donepezil was placed in the active site by aligning the 1EVE receptor to 1ACL then deleting the 1EVE receptor. A 12 Å receptor box was used for docking inhibitors into the enzyme crystal structures. We generated all molecular graphics images using the UCSF Chimera package.

The marine products aplysamine and Poly-APS were excluded from the docking analysis since aplysamine is an allosteric site inhibitor [26], and the Poly-APS are large molecular weight polymers; these compounds are not discussed in this paper. All other compounds were docked in their neutral state. The inhibitor from the crystal structure of each AChE was also docked into all AChE structures, including its own, to verify that the docking algorithm could correctly identify known crystal structure poses. Galanthamine has a conserved water molecule, to which the hydroxyl group H-bonds. Docking galanthamine into its own receptor with and without this conserved water molecule both resulted in the crystallographic pose being selected. All inhibitors generated similar scores in each receptor with the exception of donepezil in the 1ACJ receptor (Table 1). The drastic difference between donepezil in the tacrine receptor results from the Phe-330 residue being in an orientation that cannot interact with the piperidine moiety—a critical interaction for this inhibitor. Our docking results are consistent with the observed experimental data for onchidal, marinoquinoline, and tetrazacyclopentazulene compound interactions with AChE. Our data also suggest new target regions within the AChE receptor, potential structural scaffolds for novel AChE inhibitors, and reasonable binding modes for the plastoquinones and farnesylacetones. The binding scores from the docking analysis could not accurately be compared to the literature inhibition values determined experimentally since several reported values are given as IC_50_, a unit that is concentration dependent, and concentrations of enzymes used for performing the assay were not always reported in the literature. The docking scores themselves are internally consistent.

## 4. Conclusions

Exploring marine metabolites (MM) as an option for lead compounds may identify new useful interactions and scaffolds that increase potency and garner selectivity. From these compounds, we can identify new target sites within acetylcholinesterase for development of AChE-I. Future generations of AD drugs could utilize H-bonding to the oxyanion hole residues, stacking interactions with Tyr-334, and H-bonding to Tyr-121 in the PAS to inhibit AChE. Development of novel Acetylcholinesterase inhibitors that have characteristics of MM, such as an increased number of protonatable nitrogen atoms that stack against Trp-84, and creation of terpene linkers for dual site inhibitors, may help to develop compounds with greater potencies from optimization of stacking interactions in the gorge, such as the hypothetical structures shown in Figure 7. Several of these properties exhibited by the MM can also be included in the design of DFI that prevent both ACh degradation and amyloid-β aggregation. Overall these MM bind tighter to AChE than galanthamine and tacrine, and several metabolites bind tighter than donepezil. Selectively crossing scaffold features of the MM successfully produced compounds with even better AChE binding scores than the precursor scaffolds used, yet the final overall pose of some candidate compounds was not always as expected. Many compounds generated interacted with the PAS site, which is the critical interaction contributing to inhibition of amyloid-β aggregation. Determination of cholinesterase isozyme specificity (AChE *versus* butrylcholinesterase) and effects on amyloid-β aggregation for the sesquiterpene acetate [21,22], pyrrole derivative, PZT-compound, plastoquinones, and farnesylacetones classes of MM described in this article would provide an additional guide to development of novel compounds as therapies for Alzheimer’s disease.

## Supplementary Files

Supplementary File 1 Supplementary Information (PDF, 516 KB)
