# Supplementary material for: Insights and Ideas Garnered from Marine Metabolites for Development of Dual-Function Acetylcholinesterase and Amyloid-β Aggregation Inhibitors
Source: Mar Drugs. 2014 Apr 4;12(4):2114–31. doi: 10.3390/md12042114 (PMC4012451; doi:10.3390/md12042114)

Supplementary Information

Figure S1. Binding modes for tacrine, donepezil, galanthamine and rivastigmine AChE inhibitors. Modes of binding to acetylcholinesterase of current anticholinesterase drugs. (**A**) Acetylcholinesterase structure; (**B**) Tacrine; (**C**) Donepezil; (**D**) Galanthamine; (**E**) Rivastigmine. Residues highlighted are known to interact with inhibitors. Residues with purple ribbon are catalytic triad (Ser-200, Glu-327, and His-440). Cyan residues with labels represent residues having interactions to inhibitor. We generated all molecular graphics images using the UCSF Chimera package (a resource for biocomputing, visualization, and informatics at the University of California, San Francisco; supported by NIH P41 RR001081).

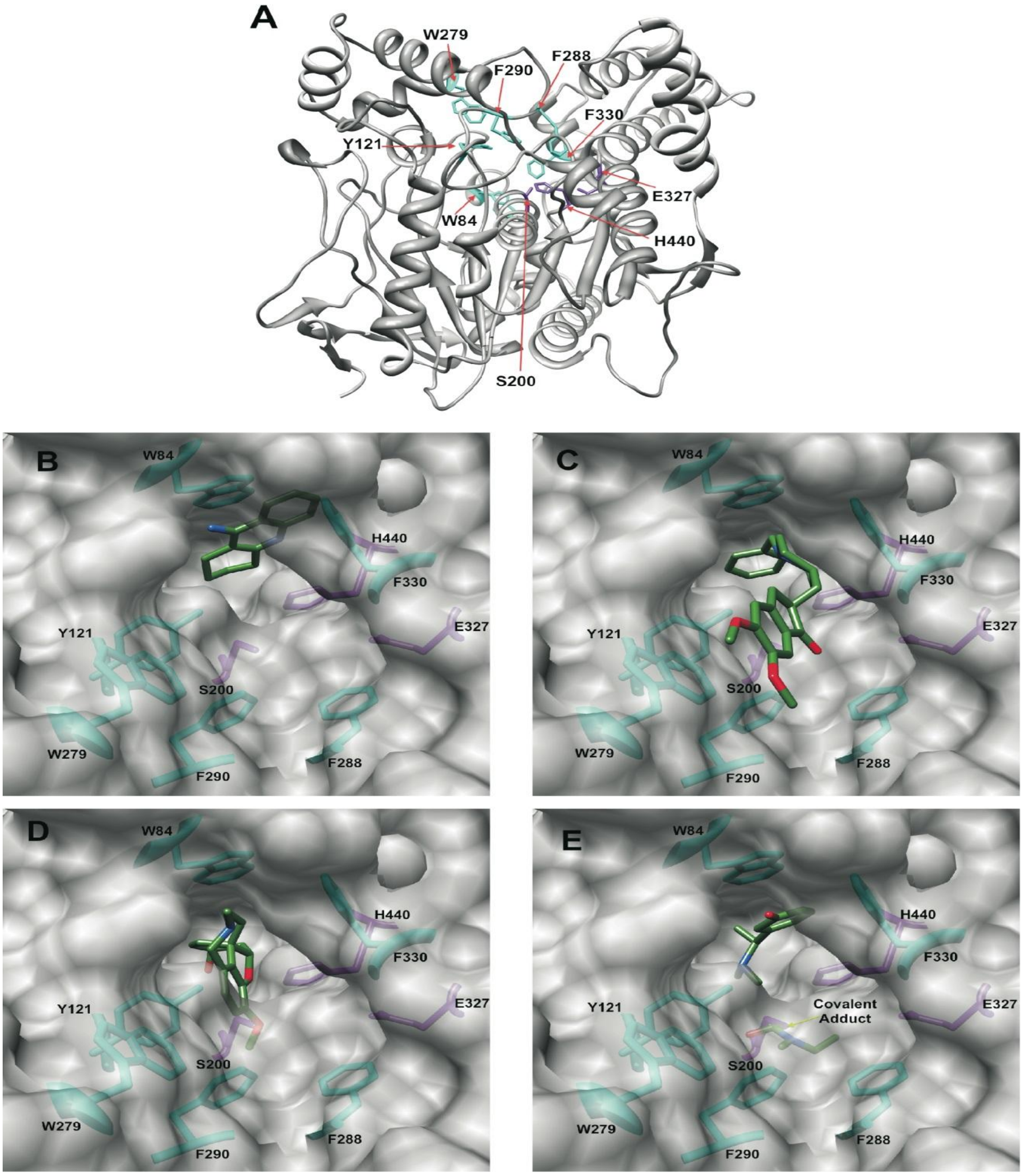

Figure S2. Docking pose of onchidal with water molecule present in 1DX6 receptor. (**A**) Onchidal poses docked in 1ACL receptor; (**B**) Onchidal pose docked in 1DX6 receptor; (**C**) Onchidal pose docked in 1EVE receptor.

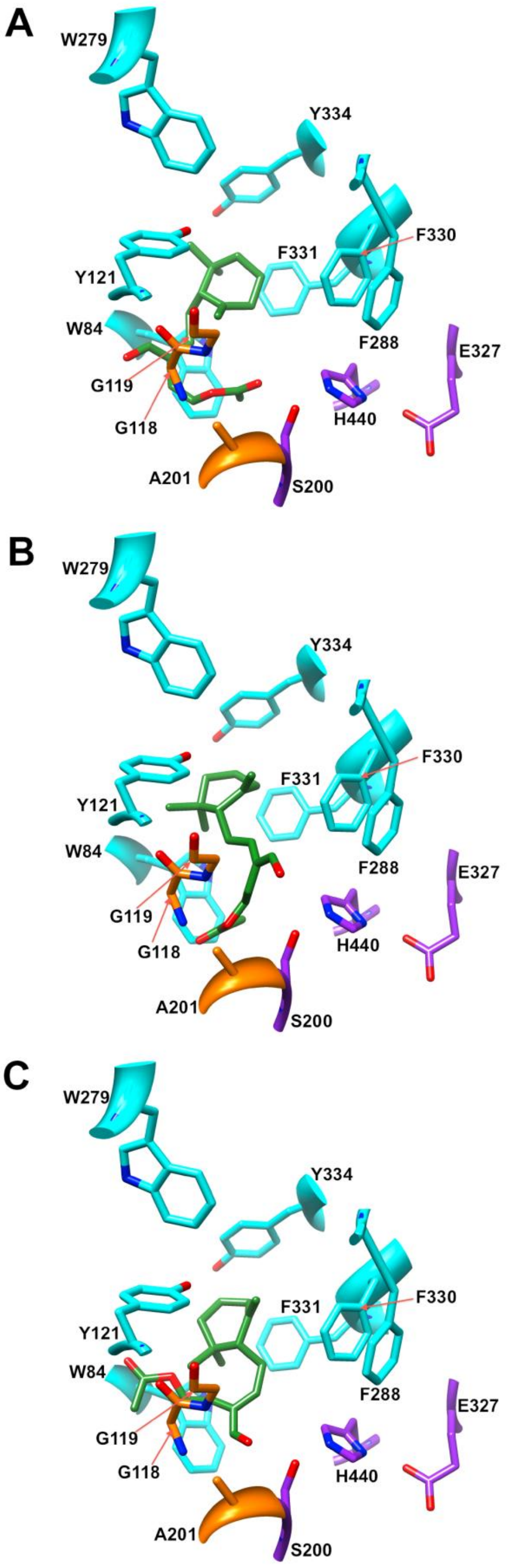

Figure S3. Docking pose of onchidal with water molecule present in 1DX6 receptor. (**A**) Overlay of onchidal poses docked with (blue) and without (green) oxyanion hole water molecule; (**B**) Onchidal pose without oxyanion water; (**C**) Onchidal with oxyanion water.

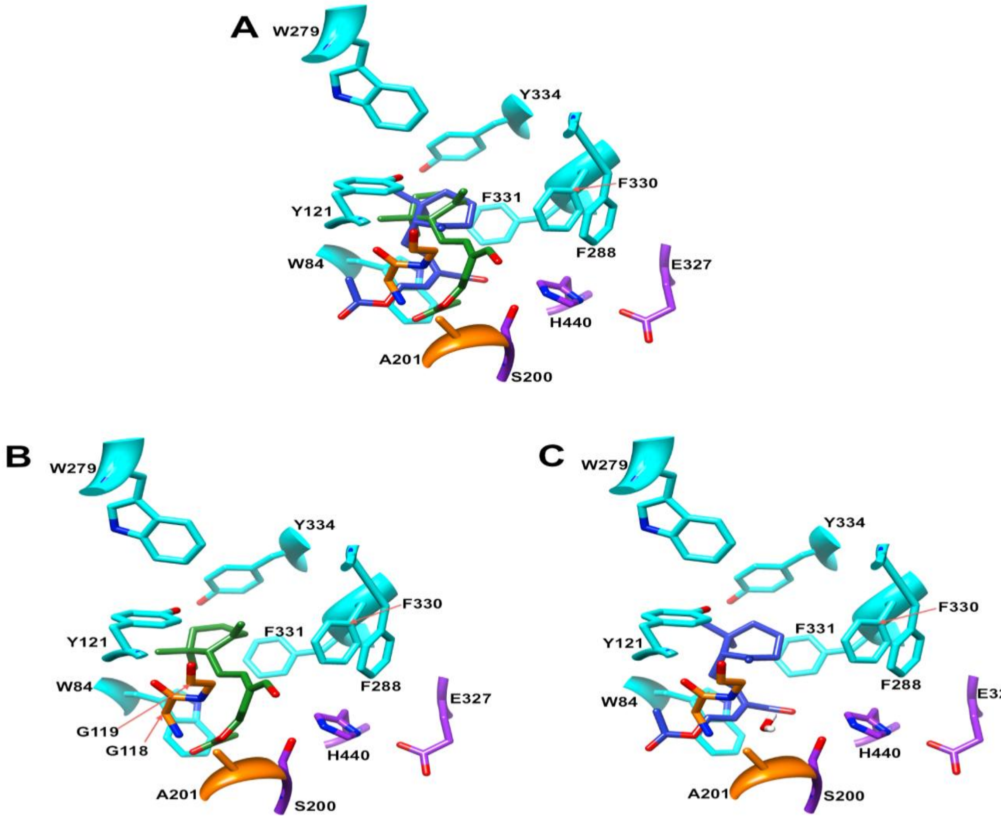

Figure S4. Alignment of 1ACJ, 1ACL, 1DX6 and 1EVE receptors. Alignment of tacrine receptor (1ACJ) places the F330 and W279 in a different conformational location than the galanthamine (1DX6) or donepezil (1EVE) or 1ACL receptors. Residues labeled in purple, orange, and cyan represent the catalytic triad, oxyanion hole, and aromatic residues of the gorge.

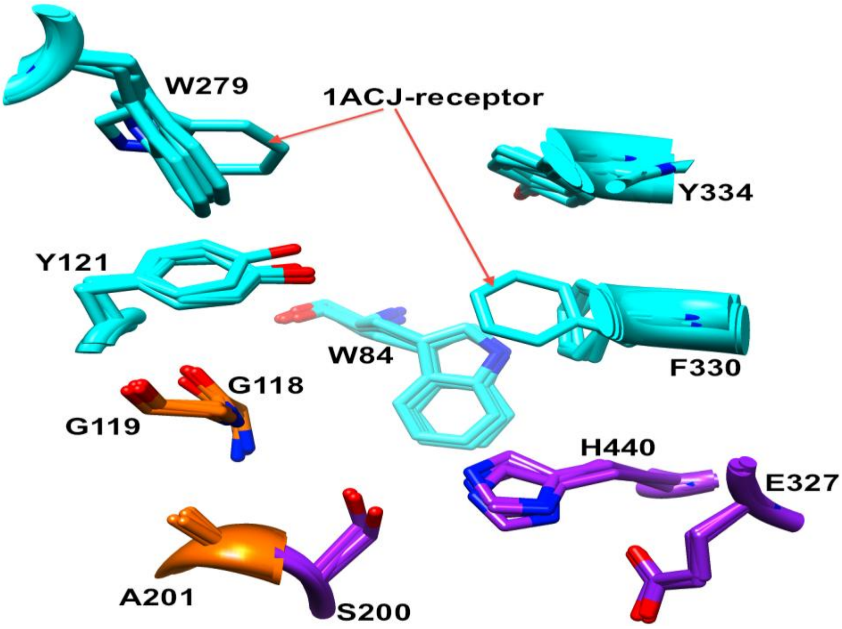

Supplement: Supplementary File 1 — Supplementary Information (PDF, 516 KB) [file marinedrugs-12-02114-s001.pdf]
